# Lipolysis by pancreatic cancer‐derived extracellular vesicles in cancer‐associated cachexia via specific integrins

**DOI:** 10.1002/ctm2.1089

**Published:** 2022-10-31

**Authors:** Chikako Shibata, Motoyuki Otsuka, Takahiro Seimiya, Takahiro Kishikawa, Kazunaga Ishigaki, Mitsuhiro Fujishiro

**Affiliations:** ^1^ Department of Gastroenterology Graduate School of Medicine The University of Tokyo Tokyo Japan; ^2^ Japan Society for the Promotion of Science Tokyo Japan

Dear Editor

Here, we report that pancreatic cancer‐derived extracellular vesicles (EVs) carry adipocyte‐targeting integrins and induce lipolysis, constituting the underlying mechanism of cancer‐associated cachexia (CAC).

CAC is a life‐threatening condition recognised as a paraneoplastic syndrome with body weight loss, skeletal mass wasting and adipose tissue atrophy.^1^ While CAC occurs in most patients with cancer,^1^ it typically manifests earlier in pancreatic cancer.^2^

Fat loss is a feature of CAC, where lipolysis is activated in adipocytes, reducing their size.^1^, ^3^ Because CAC phenotypes occur systemically, humoral factors are possibly pathogenic. However, the mechanisms underlying lipolysis remain unclear. EVs, nanoparticles released from cells into bloodstream, contain various bioactive factors that mediate intercellular communication.^4^ Cancer cells actively release EVs^5^; we also hypothesised that EVs from pancreatic cancer cells contribute to lipolysis.

Mature adipocytes were prepared from human adipose‐derived mesenchymal stem cells (Figure S1) and treated with EVs. A higher level of glycerol, a marker for lipolysis, was released from adipocytes treated with EVs from Panc‐1 and Miapaca‐2 cells, but not from Capan‐2 and Human Pancreatic Nestin‐Expressing cells (HPNE cells) (Figures 1 and S2). While cyclic adenosine monophosphate (cAMP) levels, protein kinase A (PKA) activity and the subsequent phosphorylation of hormone‐sensitive lipase (HSL) in adipocytes play key roles during lipolysis,^6^ these levels were upregulated in lipolysis‐induced adipocytes (Figure 1B,C). A reduction in lipolysis by H89, orlistat and cay10499 suggests that lipolysis, in EV‐treated adipocytes, was induced by the cAMP–PKA–HSL pathway (Figure 1D). However, cAMP levels inside EVs were similar irrespective of cell types (Figure 1E), suggesting that differential cAMP levels were not responsible for distinct lipolysis levels.

**FIGURE 1:**
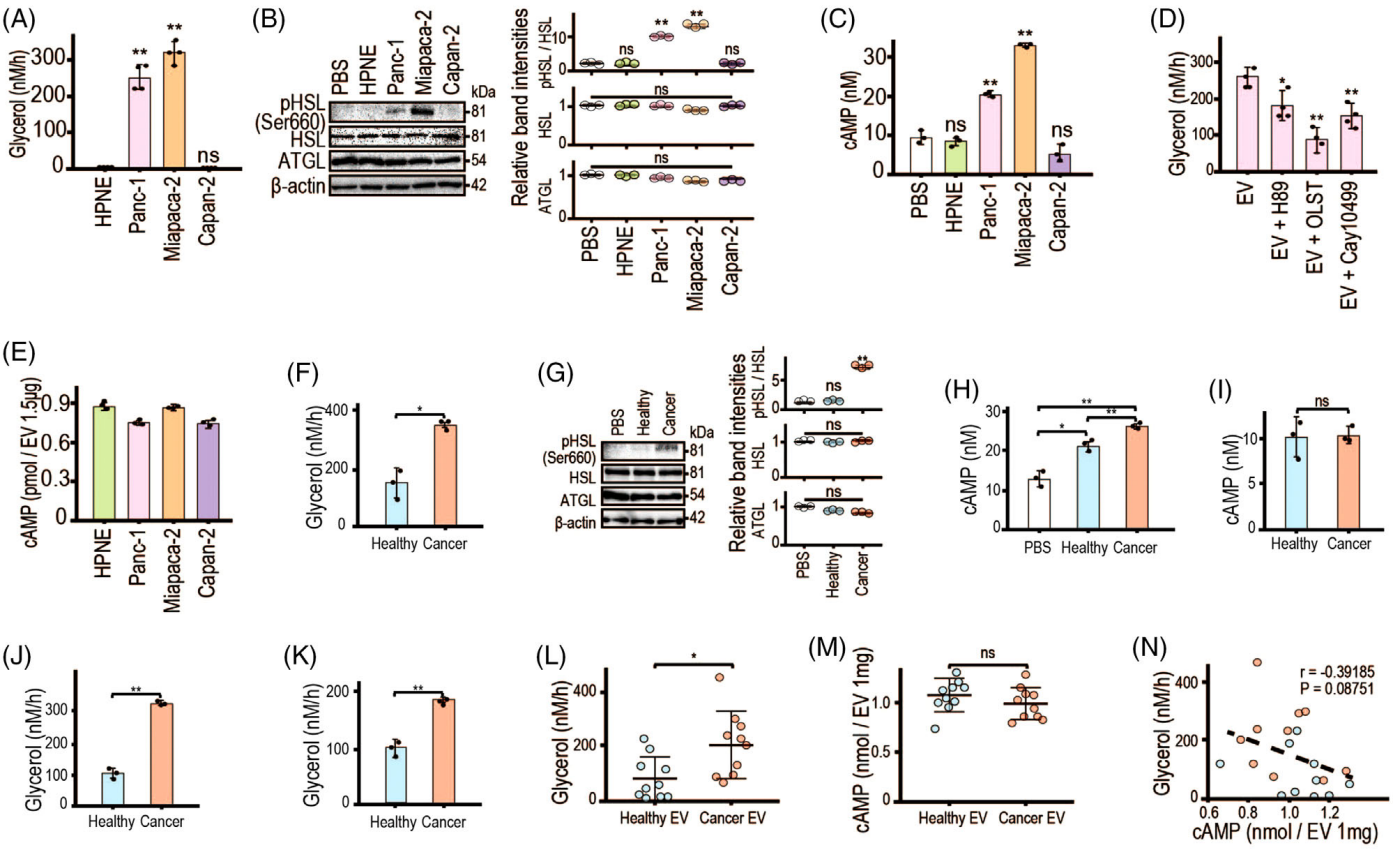
Extracellular vesicles (EVs) from pancreatic cancer induce lipolysis in human adipocytes. (A) Glycerol release from human adipocytes treated with the indicated human cell‐derived EVs (1.5 μg) for 24 h. Data are means ± standard deviations (SDs) (*n* = 4). ^**^ *p* = .0016 (Panc‐1: pancreatic cancer cell line), ^**^ *p* = .00027 (Miapaca‐2: pancreatic cancer cell line), *p* = .83 (Capan‐2: pancreatic cancer cell line). (B) Western blotting of factors related to intracellular lipolysis signaling in lysates of human adipocytes treated with the indicated cell‐derived EVs (1.5 μg) for 1 h. Representative images of three independent experiments are shown. Band intensities of phospho‐hormone‐sensitive lipase (pHSL; HSL phosphorylated at Ser660) relative to the total HSL protein level, and of total HSL and adipose trigliceride lipase (ATGL) relative to the β‐actin protein level, are shown in the right panels. Data are means ± SDs (*n* = 3). Regarding pHSL, *p* = .079 (HPNE), ^**^ *p* = .00049 (Panc‐1), ^**^ *p* < 10^−5^ (Miapaca‐2), *p* = .25 (Capan‐2); ns, not significant. (C) Cyclic adenosine monophosphate (cAMP) levels in adipocytes treated with EVs (1.5 μg) for 1 h (*n* = 3). *p* = .36 (HPNE), ^**^ *p* = .0023 (Panc‐1), ^**^ *p* = .0019 (Miapaca‐2), *p* = .11 (Capan‐2). (D) Glycerol release from human adipocytes treated with Panc‐1 cell‐derived EVs (1.5 μg) with and without 10 μM H89 (protein kinase A [PKA] inhibitor), 0.5 μM orlistat (OLST, inhibitor of HSL and ATGL), or 0.1 μM Cay10499 (HSL inhibitor). Data are means ± SDs (*n* = 4). ^*^ *p* = .030 (H89), ^**^ *p* = .00045 (OLST), ^**^ *p* = .0037 (Cay10499). (E) cAMP levels in the indicated cell‐derived EVs (1.5 μg). (F) Glycerol release from human adipocytes treated with EVs from 2.5 μl of pooled sera from five healthy controls (Healthy) or five patients with pancreatic cancer (Cancer) for 24 h. Data are means ± SDs (*n* = 3). ^*^ *p* = .032 (Healthy vs. Cancer). (G) Western blotting of factors related to intracellular lipolysis signaling in lysates of human adipocytes treated with EVs from 2.5 μl of pooled sera from five healthy controls (Healthy) or five patients with pancreatic cancer (Cancer) for 1 h. Representative images of three independent experiments are shown. Band intensities of pHSL relative to total HSL, and of total HSL and ATGL relative to β‐actin, are shown in the right panels. Data are means ± SDs (*n* = 3). Regarding pHSL, *p* = .87 (Healthy), ^**^ *p* < 10^−5^ (Cancer); ns, not significant. (H) cAMP levels in adipocytes treated with EVs from 2.5 μl of pooled sera from five healthy controls (Healthy) or five patients with pancreatic cancer (Cancer) for 1 h. Data are means ± SDs (*n* = 3). ^*^ *p* = .015 (Healthy), ^**^ *p* = .0033 (Cancer), ^**^ *p* = .0023 (Healthy vs. Cancer). (I) cAMP levels in EVs from 2.5 μl of pooled sera from five healthy controls (Healthy) or five patients with pancreatic cancer (Cancer). Data are means ± SDs (*n* = 3). ns, not significant; *p* = .82. (J and K) Glycerol release from adipocytes treated for 24 h with 10^9^ EVs (J) or with 1 μg of EVs (K) from pooled sera of five healthy controls (Healthy) or five patients with pancreatic cancer (Cancer). Data are means ± SDs (*n* = 3). ^**^ *p* < 10^−4^ (E), ^**^ *p* = .0022 (F). (L) Glycerol release from human adipocytes treated for 24 h with 1 μg of EVs from sera of healthy controls (Healthy EVs; *n* = 10) and patients with pancreatic cancer (Cancer EVs; *n* = 10). ^*^ *p* = .017. (M) cAMP levels in 1 μg of EVs derived from the serum of individual healthy controls (Healthy EVs; *n* = 10) and patients with pancreatic cancer (Cancer EVs; *n* = 10). ns, not significant; *p* = .29. (N) Correlations between glycerol release from EV‐treated adipocytes and cAMP levels in EVs. Blue and red circles indicate EVs from healthy controls (*n* = 10) and patients with pancreatic cancer (*n* = 10), respectively. The broken line is a regression line. Correlations were estimated by calculating Pearson's correlation coefficient. All the other statistical analyses were by Welch's *t*‐tests

Next, adipocytes were treated with EVs from pooled sera of healthy controls or pancreatic cancer patients. Compared with the controls, EVs from patients induced greater lipolysis (Figure 1F), and HSL phosphorylation and intracellular cAMP levels were remarkably higher (Figure 1G,H). However, the cAMP levels in EVs from controls and patients did not differ (Figure 1I). The tendency of lipolysis levels was similar even after adjustment for EV number and protein weight (Figure 1J,K). Furthermore, pancreatic cancer EVs (Table S1) induced more lipolysis (Figure 1L). However, cAMP levels did not differ between EVs from controls and patients (Figure 1M). Additionally, cAMP levels in EVs were not correlated with lipolysis levels (Figure 1N), suggesting that pancreatic cancer EVs induce greater lipolysis, possibly through a mechanism that depends on other factors.

To confirm these results, mice were intravenously injected with EVs (Figure 2A). Panc‐1‐derived EV‐treated mice exhibited 63.6% less weight gain (Figure 2B). Gonadal white adipose tissue (gWAT) was smaller in weight and size (Figure 2C,D); in addition, smaller lipid droplets were observed (Figure 2E). Consistently, EV‐treated mice exhibited increased HSL phosphorylation in the gWAT (Figure 2F). These results indicate that cancer‐derived EVs induce lipolysis via an HSL‐mediated pathway.

**FIGURE 2:**
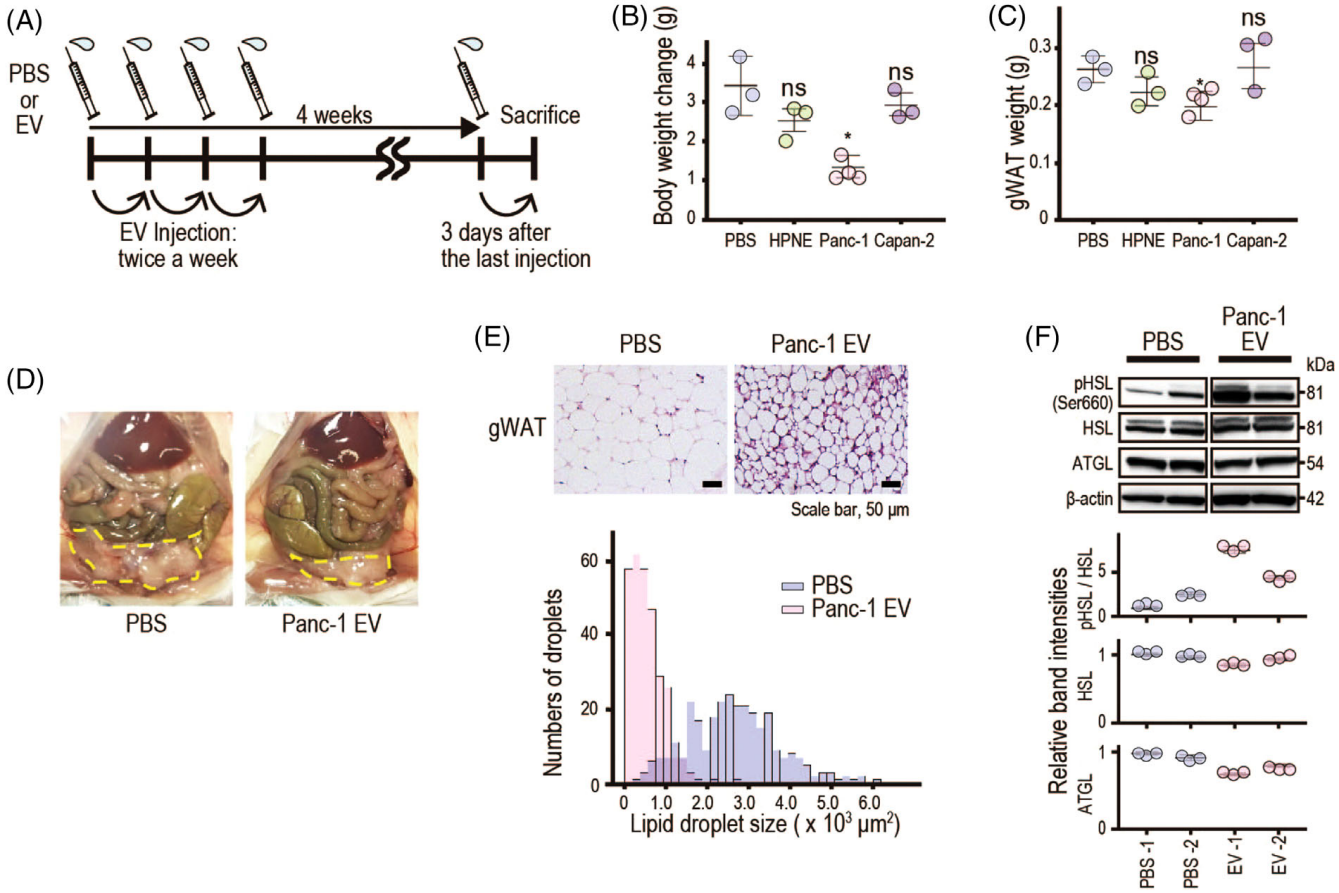
Intravenous injection of pancreatic cancer cell‐derived extracellular vesicles (EVs) induces lipolysis in vivo. (A) Schematic of the in vivo experiments. EVs (5 × 10^10^) from culture medium of HPNE, Panc‐1‐ACTB‐HiBiT and Capan‐2 cells or phosphate‐buffered saline (PBS) (control) were injected into the tail vein of BALB/c mice (6–7 weeks old, female, *n* = 3 [PBS, HPNE and Capan‐2], *n* = 4 [Panc‐1‐ACTB‐HiBiT]), twice weekly for 4 weeks. (B) Mouse body weight changes from baseline at 4 weeks after injection. Data are means ± standard deviations (SDs). ns, not significant; *p* = .13 (HPNE), ^*^ *p* = .047 (Panc‐1), *p* = .48 (Capan‐2). (C) Weight of gonadal white adipose tissue (gWAT) at 4 weeks after injection. Data are means ± SDs. ns, not significant; *p* = .21 (HPNE), ^*^ *p* = .028 (Panc‐1), *p* = .51 (Capan‐2). (D) Representative photographs of abdominal organs at 4 weeks after injection. Broken lines indicate gWAT. (E) Representative images of haematoxylin and eosin‐stained gWAT. Scale bar, 50 μm. Lipid droplet size distributions are shown in the lower panel. The sizes of 300 lipid droplets were analysed in three mice per group. (F) Western blotting of factors related to intracellular lipolysis signaling in gWAT lysates at 4 weeks after injection. Representative images from two mice per group are shown. Band intensities of phospho‐hormone‐sensitive lipase (pHSL) relative to total HSL protein, and of total HSL and ATGL relative to β‐actin protein, are shown in the lower panel. Data are means ± SDs (*n* = 3). Statistical analyses were by Welch's *t*‐test

cAMP levels in EVs were not proportional to lipolysis levels (Figure 1E,I,N). Therefore, we examined whether newly synthesised cAMP was involved in EV‐induced lipolysis. β‐Stimulators, including isoproterenol, and activate adenylyl cyclases, led to cAMP synthesis. Thus, isoproterenol induced lipolysis, which was inhibited by SQ22536, an adenylyl cyclase inhibitor. However, SQ22536 did not inhibit lipolysis induced by Panc‐1 EVs (Figure S3A,B). After 4 h, more Panc‐1‐derived EVs were taken up by EV‐treated adipocytes compared with Capan‐2 EVs (Figure S3C). Thus, EV tropism and cAMP uptake levels were linked to differential lipolysis induction.

To track EV tropism, we established reporter cells by HiBiT peptide sequence knock‐in at the *ACTB* locus in Panc‐1 cells (Panc‐1‐ACTB‐HiBiT cells) because most EVs carry β‐actin^7^ (Figure S4). EVs were taken up by adipocytes and lung tissue, not muscle or liver tissues (Figure 3A). As integrin patterns may be associated with EV tropism,^8^ integrin expression profiles in Panc‐1 cell‐derived EVs were evaluated (Figure 3B). Integrins α_6_ (ITGA6), β_1_ (ITGB1) and α_V_ (ITGAV) are dominantly expressed in Panc‐1‐ and Miapaca‐2‐derived EVs. Remarkably, ITGB1 was not expressed in EVs derived from Capan‐2, which did not induce lipolysis (Figure 3C). Thus, we hypothesised that ITGB1‐related integrin heterodimers play a key role in EV‐induced lipolysis. ITGB1 pairs with ITGA6 (integrin α_6_β_1_) to generate classical ‘laminin‐specific’ integrins, laminin α5 (LAMA5) and α4 (LAMA4), which are expressed in adipose and lung tissues, not skeletal muscle or liver tissues in mice (Figure 3D,E). Similarly, laminin subfamilies were highly expressed in human adipose and lung tissues (Figure 3F). Thus, interactions between integrins and laminin may determine the tropism of pancreatic cancer‐derived EVs to adipocytes.

**FIGURE 3:**
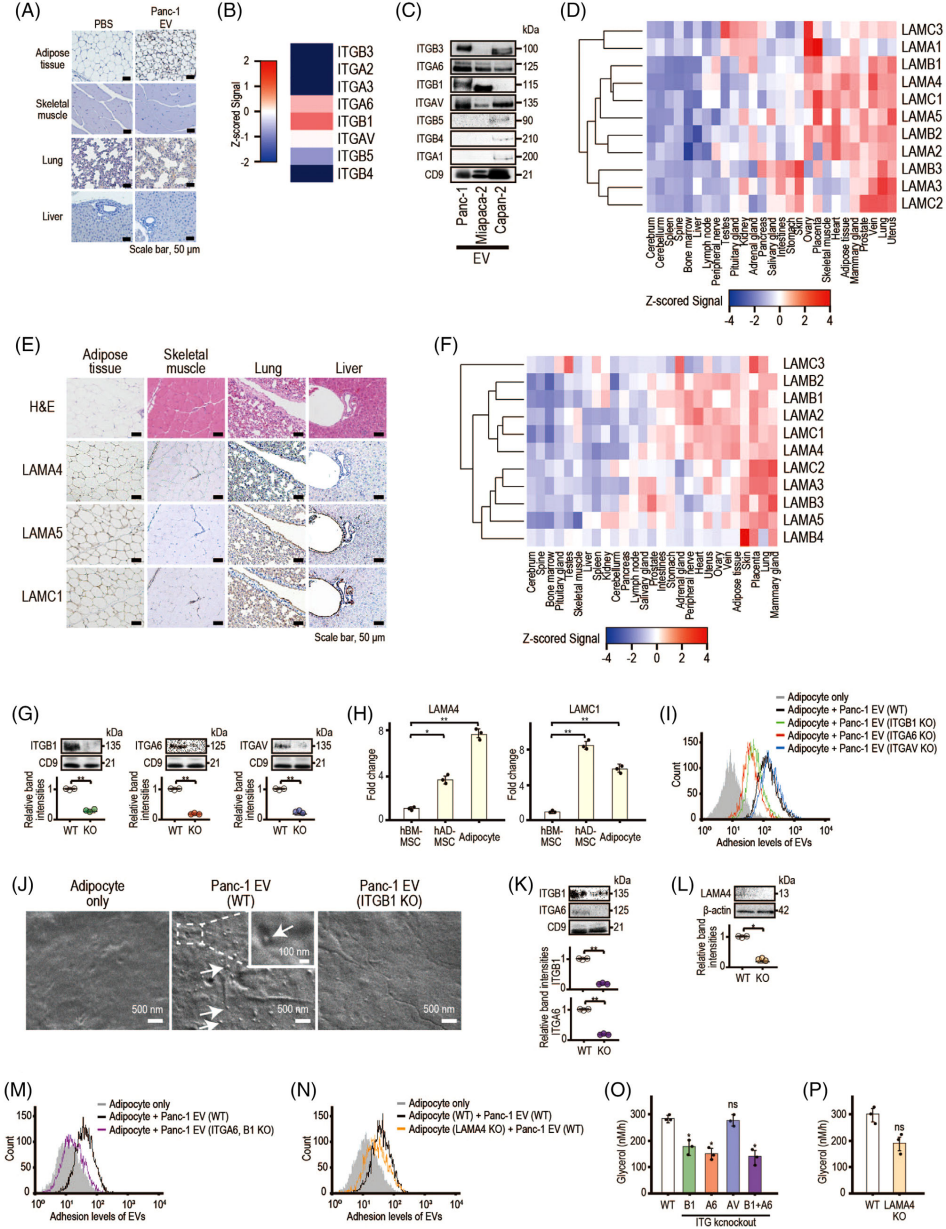
Panc‐1 cell‐derived extracellular vesicles (EVs) preferably distribute to adipose and lung tissues via ITGB1 and ITGA6. (A) Representative images of immunohistochemistry findings, using an anti‐HiBiT antibody for analysis of the indicated tissues from mice treated with phosphate‐buffered saline (PBS) or Panc‐1 cell‐derived EVs containing ACTB‐HiBiT for 4 weeks. Scale bar, 50 μm. (B) Expression levels of integrin subfamilies in Panc‐1 cell‐derived EVs. Data from the ProteomeXchange Consortium Database (PXD018301). (C) Western blotting of integrin subfamilies in EVs derived from the indicated cells. Representative images of three independent experiments are shown. (D) Heatmap of the expression levels of laminin subfamilies in mouse tissues based on *Z*‐scores. Data from the RefEx Database. (E) Representative images of immunohistochemistry findings, using anti‐laminin α4 (LAMA4), anti‐laminin α5 (LAMA5) and laminin γ1 (LAMC1) antibodies for analysis of the indicated tissues from 10‐week‐old female BALB/c mice. Scale bar, 50 μm. (F) Heatmap of the expression levels of laminin subfamilies in human tissues based on *Z*‐scores. Data from the RefEx Database. (G) Western blotting to confirm the knockout of each integrin in Panc‐1 cell‐derived EVs. Representative images of three independent experiments are shown. Band intensities of each integrin relative to CD9 are shown in the lower panel. Data are means ± standard deviations (SDs) (*n* = 3). ^**^ *p* = .00016 (ITGB1), ^**^ *p* = .00039 (ITGA6), ^**^ *p* = .00013 (ITGAV). (H) Expression levels of laminin α4 (LAMA4) and laminin γ1 (LAMC1) in differentiated adipocytes, as determined by Reverse transcription‐quantitative polymerase chain reaction (RT‐qPCR). Data are means ± SDs (*n* = 3). ^*^ *p* = .013 (LAMA4, human adipose‐derived mesenchymal stem cells [hAD‐MSC]), ^**^ *p* = .0024 (LAMA4, adipocyte), ^**^ *p* = .00082 (LAMC1, hAD‐MSC), ^**^ *p* = .0024 (LAMC1, adipocyte). (I) Flow cytometry analysis of adhesion to adipocytes among EVs derived from wild‐type (WT), ITGB1‐knockout (ITGB1 KO), ITGA6‐knockout (ITGA6 KO) and ITGAV‐knockout (ITGAV KO) Panc‐1 cells. Representative images of three independent experiments are shown. (J) Electron microscopy using the NanoSuit method. Human adipocytes were treated for 4 h with EVs derived from WT or ITGB1 KO Panc‐1 cells. (K) Western blotting was performed to confirm the double knockout of ITGA6 and ITGB1 in Panc‐1 cell‐derived EVs. Representative images of three independent experiments are shown. Band intensities of each integrin relative to CD9 are shown in the lower panel. Data are means ± SDs (*n* = 3). ^**^ *p* = .00033 (ITGB1), ^**^ *p* = .0017 (ITGA6). (L) Western blotting was performed to confirm the knockout of LAMA4 in differentiated human adipocytes. Representative images of three independent experiments are shown. Band intensities of each integrin relative to β‐actin are shown in the lower panel. Data are means ± SDs (*n* = 3). ^**^ *p* = .018. (M) Flow cytometry analysis about the adhesion to WT adipocytes by EVs derived from WT or ITGA6‐ and ITGB1‐double knockout Panc‐1 cells. Representative images of three independent experiments are shown. (N) Flow cytometric analysis of the extent of adhesion of EVs derived from WT Panc‐1 cells to WT or laminin α4‐knockout (LAMA4 KO) adipocytes. Representative images of three independent experiments are shown. (O) Glycerol release from human adipocytes treated with the indicated Panc‐1 cell‐derived EVs (1.5 μg) for 24 h. Data are means ± SDs (*n* = 3). ^*^ *p* = .015 (ITGB1 KO), ^*^ *p* = .040 (ITGA6 KO), *p* = .77 (ITGAV KO), ^*^ *p* = .041 (ITGB1 and ITGA6 KO). (P) Glycerol release from WT human adipocytes and LAMA4‐knockout human adipocytes treated with Panc‐1 cell‐derived EVs (1.5 μg) for 24 h. Data are means ± SDs (*n* = 3). ns, not significant; *p* = .058 (LAMA4 KO). Statistical analyses were by Welch's *t*‐test

We isolated ITGB1‐knockout, ITGA6‐knockout and ITGAV‐knockout EVs to evaluate the roles of ITGB1 and ITGA6 in lipolysis, through establishing Panc‐1 and Miapaca‐2 cells lacking these molecules or an unrelated ITGAV as a control (Figures 3G and S5A). After confirming that laminin was expressed in human adipocytes (Figure 3H), we determined the level of interaction between adipocytes and EVs. ITGB1‐knockout and ITGA6‐knockout EVs demonstrated reduced interactions with adipocytes (Figures 3I and S5B); thus, ITGB1 and ITGA6 are crucial for adipocyte–EV interactions. Many EVs were present on the adipocyte surface after treatment (Figure 3J). Several wild‐type EVs derived from Panc‐1 cells are ingested by adipocytes. Double knockout of ITGA6 and ITGB1 on EVs or LAMA4‐knockout adipocytes showed reduced interactions between EVs and adipocytes (Figure 3K–N) and lipolysis was suppressed proportionally (Figures 3O,P and S5C), suggesting that ITGB1 and ITGA6 on pancreatic cancer‐derived EVs determine EV tropism to adipocytes.

As reported,^9^ the number of EVs was higher in patients’ sera, and their size was smaller (Figure 4A,B), confirming TSG101 and CD63 expression (Figure 4C). Cancer‐derived CA19‐9‐positive EVs or CD63‐positive were specifically concentrated (Figure 4D–F). ITGB1 and ITGA6 expression levels in bulk sera or CD63‐positive EVs were not significantly different between controls and patients. However, positivity rates for ITGB1 and ITGA6, but not ITGAV, were significantly higher in concentrated cancer‐derived EVs (Figure 4G).

**FIGURE 4:**
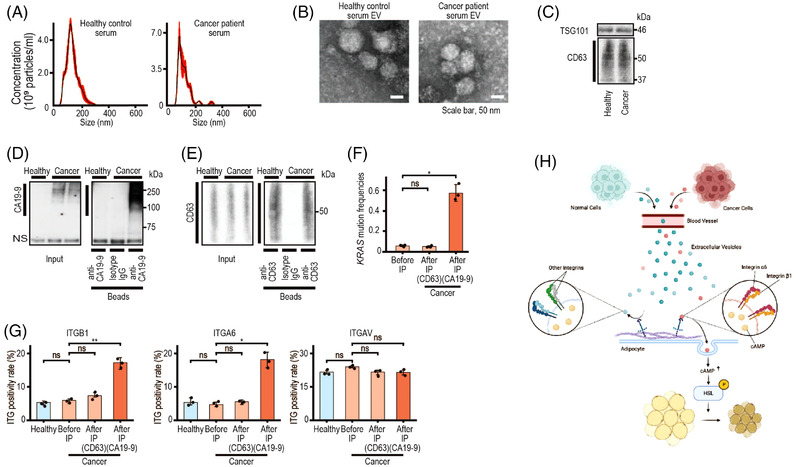
Pancreatic cancer‐derived extracellular vesicles (EVs) in patients’ sera have higher ITGB1 and ITGA6 levels. (A) Size distributions of EVs from pooled sera of five healthy controls or five patients with pancreatic cancer. (B) Transmission electron micrographs of EVs from pooled sera of the indicated groups. Representative images of at least three independent experiments are shown. Scale bar, 50 nm. (C) Western blotting of exosome marker levels (TSG101 and CD63) in 1.5 μg of EVs from pooled sera of the indicated groups. (D and E) Isolation of pancreatic cancer‐derived EVs from serum by immunoprecipitation (IP) targeting CA19‐9 (D) or CD63 (E). EVs from pooled sera of five healthy controls (Healthy) or five patients with pancreatic cancer (Cancer) were subjected to IP. Aliquots of each sample before IP (2% for CA19‐9 and 20% for CD63) were used as the input. An anti‐CD63 and an anti‐CA19‐9 antibody were used for IP; an isotype IgG was used as the negative control. Representative western blotting images of three independent experiments are shown. NS, nonspecific band. (F) Droplet digital polymerase chain reaction (ddPCR) analysis of *KRAS* mutation frequencies in RNAs in EVs from pooled sera of patients with pancreatic cancer (Before IP) or EVs captured by anti‐CD63 and anti‐CA19‐9 beads from pooled sera. Data are means ± standard deviations (SDs) (*n* = 3). ns, not significant; *p* = .38 (CD63), ^*^ *p* = .026 (CA19‐9). (G) Integrin positivity rate on EVs from pooled sera of five healthy controls (Healthy), bulk EVs from pooled sera of patients with pancreatic cancer (Before IP), and EVs specifically captured by anti‐CD63 and anti‐CA19‐9 beads. The positivity rate was analysed by NanoSight after EVs had been fluorescently labelled with the indicated integrin antibodies. The positivity rate in bulk EVs from cancer patients (Before IP) were compared with the rate of other groups of EVs. ns, not significant; *p* = .23 (ITGB1, Healthy), *p* = .13 (ITGB1, CD63), ^*^ *p* = .0064 (ITGB1, CA19‐9), *p* = .72 (ITGA6, Healthy), *p* = .27 (ITGA6, CD63), ^*^ *p* = .016 (ITGA6, CA19‐9), *p* = .071 (ITGAV, Healthy), *p* = .21 (ITGAV, CD63), ^*^ *p* = .22 (ITGAV, CA19‐9). Statistical analyses were by Welch's *t*‐test. (H) Summary of this study. Pancreatic cancer‐derived EVs induce lipolysis by cyclic adenosine monophosphate (cAMP) in EVs. EVs derived from normal cells induce lipolysis only slightly because these EVs are rarely captured by adipocytes. In contrast, pancreatic cancer‐derived EVs are actively captured through specific integrins on these EVs. Therefore, pancreatic cancer induces higher levels of lipolysis, one of the phenotypes of cachexia

In conclusion, we propose the importance of ITGB1 and ITGA6 expression in cancer‐derived EVs for lipolysis induction during CAC (Figure 4H) in addition to the importance of examining tissue‐specific EVs in a heterogeneous EV population in sera.^10^

## CONFLICT OF INTEREST

The authors declare they have no conflicts of interest.

## Supporting information

Supporting Information
