# Supplementary material for: Lipolysis by pancreatic cancer‐derived extracellular vesicles in cancer‐associated cachexia via specific integrins
Source: Clin Transl Med. 2022 Oct 31;12(11):e1089. doi: 10.1002/ctm2.1089 (PMC9619371; doi:10.1002/ctm2.1089)
Supplement: Supplementary file 1 — Supporting Information [file CTM2-12-e1089-s001.docx]

**Supporting Information**

## Lipolysis by Pancreatic Cancer-Derived Extracellular Vesicles in Cancer- Associated Cachexia via Specific Integrins

Chikako Shibata, Motoyuki Otsuka, Takahiro Seimiya, Takahiro Kishikawa, Kazunaga Ishigaki, and Mitsuhiro Fujishiro

**Guide to the Supporting Information Supplementary Text Supplementary Methods Supplementary References**

**Supplemental Tables (Supplemental Tables S1 and S2) Supplemental Figures (Supplemental Figures S1-7)**

**SUPPLEMENTARY TEXT**

**Isolation method of pancreatic cancer-derived EVs**

We hypothesized that pancreatic cancer-derived EVs have higher levels of ITGB1 and ITGA6. Investigation of this hypothesis required the specific isolation of cancer-derived EVs from the heterogenous EVs in serum. To achieve this, we focused on carbohydrate antigen 19-9 (CA19-9), a sugar chain on the cell surface ^1^ and a marker of pancreatic cancer. Although CA19-9 is produced in normal pancreatic epithelial cells, its synthesis is substantially increased by aberrant sialylation in pancreatic cancer cells ^1^. Because EVs are produced by cell membrane recycling ^2^, we hypothesized that CA19-9 is present on the surface of EVs from CA19-9-positive cells. As expected, CA19-9 was abundant in bulk sera of pancreatic cancer patients and in the EVs therein (Figure S6A-C). Consistently, it was confirmed that CA19-9 levels in EVs were in proportion to its expression levels in cells, determined by using normal human pancreatic ductal epithelial cells (HPDE and HPNE), CA19-9-negative pancreatic cancer cells (Panc-1 and Miapaca- 2), and CA19-9-positive pancreatic cancer cells (BxPC-3 and Capan-2) (Figure S6D-G). To establish a method for isolation of CA19-9-positive EVs, we first prepared unrelated EVs from Huh7 hepatocellular carcinoma cells (Figure S7A). Huh7 cell- derived EVs (CA19-9-negative), Capan-2 cell-derived EVs (CA19-9-positive), and a mixture of these EVs were immunoprecipitated (IP) by targeting CA19-9. Western blotting of IP samples confirmed the isolation of highly concentrated CA19-9-positive EVs. Specificity was confirmed by using an isotype IgG for IP and by assaying AFP (i.e., an abnormal protein used as a marker of hepatocellular carcinoma), which was present

only in Huh7 cell-derived EVs (Figure S7B).

To verify the specificity further, *KRAS* mutation frequencies in mRNAs in the

EVs were examined, because pancreatic cancer cells typically have *KRAS* mutation alleles ^3^. We examined *KRAS* mutation frequencies in the input samples (before IP) and IP samples (after IP) from equal number mixtures of Huh7 (carrying only wild-type *KRAS*) cell-derived and Capan-2 (carrying mutant *KRAS* in hetero) cell-derived EVs (Figure S7C). After confirming that preamplification did not affect the results (Figure S7D), we found that *KRAS* mutation frequencies were significantly higher in the IP samples (After IP) than the input samples (Before IP) (Figure S7E), indicating high concentration of CA19-9 positive Capan-2 cell-derived EVs. These results confirmed that pancreatic cancer-derived EVs can be concentrated by IP targeting CA19-9-positive EVs.

## SUPPLEMENTARY METHODS

**EV-TRACK deposition**

We have deposited the EV experimental data in the EV-TRACK knowledgebase ^4^ (EV- TRACK ID: EV210250).

## Human serum samples

Serum samples were obtained from patients with pancreatic ductal adenocarcinoma who were hospitalized or had attended the outpatient clinic at the University of Tokyo Hospital from April 2020 to July 2021. The diagnosis of pancreatic ductal adenocarcinoma was pathologically confirmed using tissues that had been obtained by fine-needle aspiration or on the basis of characteristic findings in computed tomography and magnetic resonance cholangiopancreatography examinations. Control samples were obtained from age- matched cancer-free patients (e.g., individuals with fatty liver, gastro-esophageal reflux disease, or chronic gastritis) during the same period.

## Cell culture

Panc-1 cells were purchased from the European Collection of Authenticated Cell Cultures (Salisbury, UK). Miapaca-2 cells, human adipose-derived mesenchymal stem cells (hAD- MSCs), and human bone marrow-derived mesenchymal stem cells were purchased from the Japanese Collection of Research Bioresources (Osaka, Japan). 293T, HPDE, HPNE, BxPC-3, Capan-2, and Huh7 cells were purchased from the American Type Culture Collection (Manassas, VA, USA). Panc-1 cells, hAD-MSCs, and human bone marrow- derived mesenchymal stem cells were cultured in Dulbecco’s modified Eagle’s medium (DMEM), high glucose, supplemented with 10% fetal bovine serum (FBS) and 1%

GlutaMAX (Thermo Fisher Scientific, Waltham, MA, USA). 293T, HPNE, Miapaca-2, and Huh7 cells were cultured in DMEM, low glucose, supplemented with 10% FBS. HPDE cells were cultured in Keratinocyte Growth Medium 2 supplemented with the SupplementPack (TaKaRa Bio, Shiga, Japan). BxPC-3 and Capan-2 cells were cultured in Roswell Park Memorial Institute 1640 Medium supplemented with 10% FBS. All cells were incubated at 37°C in an atmosphere that contained 20% O2 and 5% CO2.

## Reagents, stimulators, and inhibitors

Orlistat was obtained from Tokyo Chemical Industry (Tokyo, Japan). H89 and SQ22536 were purchased from Selleck Biotech (Tokyo, Japan). Cay10499 was obtained from Cayman Chemical (Ann Arbor, MI, USA). Human adipocytes were pretreated with these inhibitors for 15 min before stimulation. Isoproterenol was purchased from Wako.

## Isolation and characterization of EVs

Cells were cultured in serum-free medium before isolation of EVs. For HPNE, Panc-1, Miapaca-2, and Huh7 cells, serum-free medium comprised advanced DMEM (Thermo Fisher Scientific) supplemented with 2% GlutaMAX. For BxPC-3 and Capan-2 cells, serum-free medium comprised advanced Roswell Park Memorial Institute 1640 Medium (Thermo Fisher Scientific) supplemented with 2% GlutaMAX. For HPDE cells, serum- free medium comprised normal Keratinocyte Growth Medium 2 supplemented with the SupplementPack. After incubation for 72 h, conditioned media were centrifuged at 2000

× g for 20 min at 4°C to remove cellular debris. The supernatants were concentrated by ultrafiltration using an Amicon Ultra-15 100K (Merck Millipore, Billerica, MA, USA). qEV size exclusion chromatography columns (single 35) with an automated fraction

collector (Izon Science, Christchurch, New Zealand) were used for EV isolation, in accordance with the manufacturer’s instructions. Briefly, the column was loaded into the automated fraction collector and flushed with 12 mL of PBS before sample loading. Aliquots of concentrated culture medium (150 μL each) were loaded onto the column and flushed with PBS. The automated fraction collector was programmed to discard the first 1 mL and collect the subsequent 0.6 mL as an EV-containing fraction. When isolating EVs from human sera, frozen stock sera were thawed and centrifuged at 2000 × g for 10 min at 4°C, then passed through a 0.45-μm filter (Merck Millipore) to remove debris. Next, 150 μL of the samples were loaded onto the qEV size exclusion chromatography columns. The EVs were enumerated and sized using the NanoSight LM10 (Malvern Instruments, Malvern, UK). EV protein weight was determined by the Bio-Rad Protein Assay (Bio-Rad Laboratories, Hercules, CA, USA) or the Qubit Protein Assay (Thermo Fisher Scientific). To visualize EVs, transmission electron microscopy was performed at the Hanaichi UltraStructure Research Institute (Aichi, Japan). Exosome markers such as CD63 and TGS101 were evaluated by Western blotting.

## Differentiation of hAD-MSCs into human adipocytes

Differentiation of human adipocytes from hAD-MSCs was induced as previously reported ^5^. Briefly, hAD-MSCs were seeded onto six-well plates and cultured in DMEM high glucose supplemented with 10% FBS, 1% GlutaMAX, 0.5 mM 3-isobutyl-1- methylxanthine (IBMX), 10 μg/mL insulin, 1 μM dexamethasone, and 100 μM indomethacin. IBMX, insulin, dexamethasone, and indomethacin were purchased from Wako (Osaka, Japan). The medium was changed at 3-day intervals for 21 days. Differentiated human adipocytes were used 21–28 days after the start of induction.

## Oil red O staining of adipocytes

Oil red O powder (Wako) was dissolved in 99% 2-propanol (Wako) to a final concentration of 0.3%. The resulting solution and distilled water were mixed at a ratio of 3 to 2; the mixture was passed through a 0.45-μm filter (Merck Millipore) after incubation at room temperature for 30 min. This solution was used as Oil red O solution. Induced adipocytes were fixed in 2% paraformaldehyde for 10 min, washed with 60% 2-propanol for 1 min, and stained with Oil red O solution for 20 min at room temperature. After cells had been washed with deionized distilled water, they were mounted in Mount-Quick Aqueous solution (Daido Sangyo, Saitama, Japan). Images were captured using the DP72 microscope digital camera system with DP2-TWAIN software (Olympus, Tokyo, Japan). To quantify intracellular lipids, 1 × 10^5^ hAD-MSCs and adipocytes were seeded onto 96- well plates. Cells were stained with Oil red O as described above and dried in air overnight. Subsequently, cells were washed with 60% 2-propanol, then incubated with 200 μL of 4% Triton X-100 in 2-propanol for 3 min. After incubation, the supernatant (100 μL) in each well containing Oil red O eluted from adipocytes was transferred into a new 96-well plate; the absorbance at 650 nm was measured using a Multiskan FC (Thermo Fisher Scientific).

## Cellular RNA extraction and reverse transcription (RT)-quantitative polymerase chain reaction (qPCR) analysis

Cellular mRNAs were extracted using Isogen Ⅱ (Nippon Gene, Tokyo, Japan). Reverse transcription was performed using ReverTra Ace qPCR RT Master Mix (Toyobo, Osaka, Japan). qPCR was performed using the Thunderbird SYBR qPCR Mix (Toyobo) and

StepOnePlus (Thermo Fisher Scientific), in accordance with the manufacturer’s instructions. Expression levels were normalized relative to glyceraldehyde-3-phosphate dehydrogenase (GAPDH) using the ΔΔCT method (ΔΔCT = ΔCTsample − ΔCTGAPDH). The primer sequences are listed in Supplemental Table 2.

## EV labeling and internalization

EVs were fluorescently labeled using the ExoSparkler Exosome Membrane Labeling Kit– Green (Dojindo, Kumamoto, Japan) in accordance with the manufacturer’s instructions; this method enables EV membranes to be labeled without self-particle formation or a shift in EV size. Labeled EVs were added to human adipocyte culture medium for 5 h at 37℃. After cells had been washed in PBS, they were mounted in VECTASHIELD Mounting Medium with DAPI (Vector Laboratories, Burlingame, CA, USA). Intracellular fluorescence was observed using the Olympus DP72 microscope digital camera system.

## HiBiT peptide sequence knock-in by gene editing

HiBiT peptide sequences were knocked-in at the 3ʹ-terminus of the ACTB gene locus in Panc-1 cells via the CRISPR-Cas9 method. Briefly, recombinant Cas9 protein, guide RNA (tracrRNA + crRNA: 5ʹ-CGTCCACCGCAAATGCTTCT-3ʹ), and donor oligonucleotides

(5ʹ-CCTGGCCTCGCTGTCCACCTTCCAGCAGATGTGGATCAGCAAGCAGGAG TATGACGAGTCCGGCCCCTCCATCGTCCACCGCAAATGCTTCGTGAGCGGCT GGCGGCTGTTCAAGAAGATTAGCTAGTCGGACTATGACTTAGTTGCGTTACA CCCTTTCTTGACAAAACCTAACTTGCGCAGAAAACAAGATGAGATTG-3ʹ

[Integrated DNA Technologies, Coralville, IA, USA]) were transfected into the cells

using Lipofectamine RNAiMAX reagent (Thermo Fisher Scientific), in accordance with the manufacturer’s instructions. Diluted cells were seeded onto 48-well plates and their supernatants were subjected to HiBiT luciferase assays to identify successfully knocked- in cells. After the analysis of HiBiT protein in culture medium, polyclonal cells with higher luciferase activities were selected as knocked-in cells (Panc-1-ACTB-HiBiT cells). To confirm knock-in success, Western blotting of cell lysates was performed using the Nano Glo HiBiT blotting system (Promega, Madison, WI, USA). High luciferase values in cell lysates and EVs derived from cells were confirmed by HiBiT luciferase assays.

## HiBiT luciferase assay

The presence of HiBiT peptides conjugated with β-actin protein in cells, culture medium, and EVs from the cells was determined using the Nano Glo HiBiT Lytic Detection System (Promega). Briefly, samples were mixed with HiBiT lysis buffer, then incubated with recombinant LgBiT protein and the luciferase substrate for 10 min. LgBiT protein readily binds to HiBiT peptides, producing active nano-luciferases. Luciferase activities were assayed using the GloMax Multi-Detection System (Promega).

## Assessment of lipolysis

The magnitude of lipolysis was estimated by determining the glycerol level in culture medium. Human adipocytes were treated with EVs in phenol red-free DMEM (Wako) supplemented with 0.5% bovine serum albumin (Wako) for 24 h. The glycerol level in culture medium was assayed using a Glycerol Assay Kit (Sigma-Aldrich, St. Louis, MO, USA), in accordance with the manufacturer’s instructions. The absorbance at 650 nm was measured using a Multiskan FC (Thermo Fisher Scientific). For displaying the results,

the baseline lipolysis levels from controls (treated with PBS) were subtracted from those of samples in each assay to adjust the variables of baseline lipolysis levels.

## Western blotting analysis and antibodies

Cells were homogenized in lysis buffer (50 mM Tris [pH 8.0], 150 mM NaCl, 0.1% sodium dodecyl sulfate, 1% NP40, 0.5% sodium deoxycholate, and 0.02% sodium azide) for 15 min on ice. Homogenates were centrifuged at 15,000 rpm for 10 min; supernatants were then collected. Sample buffer (4×) was added to the supernatant (final concentration, 62 mM Tris [pH 6.8], 2% sodium dodecyl sulfate, 5% 2-mercaptoethanol, and 10% glycerol) and the mixture was boiled at 95°C for 5 min. EVs were lysed in 4× sample buffer for 15 min on ice and boiled at 95°C for 5 min. For detection of CD9 and CD63, EVs were lysed under non-reducing conditions using sample buffer without 2- mercaptoethanol; they were not boiled. Samples were separated by sodium dodecyl sulfate–polyacrylamide gel electrophoresis in polyacrylamide gels (Wako), followed by electrophoretic transfer onto polyvinylidene fluoride membranes (GE Healthcare, Waukesha, WI, USA). After membranes had been blocked with 5% dry milk, they were probed with the appropriate primary antibodies (indicated below) diluted in Immunoshot Reagent 1 (Cosmo Bio, Tokyo, Japan) overnight at 4°C. The corresponding horseradish peroxidase-conjugated secondary antibodies (GE Healthcare) were subsequently added. For detection of CD9 and CD63, primary antibody incubation was performed immediately after transfer; the blocking step was omitted. For detection of CA19-9, an anti-mouse IgM horseradish peroxidase-conjugate (Tokyo Chemical Industry) was used as the secondary antibody because the primary antibody was mouse IgM. Bound antibodies were detected using ImmunoStar LD (Wako). Band intensities were analyzed

using ImageJ software (National Institutes of Health, Bethesda, MD, USA). The following primary antibodies were used: TSG101 (NB200-11) antibody (Novus Biologicals, Centennial, CO, USA); CD9 (014-27763) and CD63 (012-27063) antibodies (Wako); phospho-HSL Ser660 (PA5-110131) antibody (Thermo Fisher Scientific); HSL (#3991), ATGL (#2138), AFP (#4448), integrin β3 (#13166), integrin α6 (#3750),

integrin β1 (#4706), integrin αV (#4711), integrin β5 (#3629), integrin β4 (#14803), integrin α1 (#71747), and β-actin (#5125) antibodies (Cell Signaling Technology, Danvers, MA, USA); LAMA4 (10465-1-AP) antibody (Proteintech, Rosemont, IL, USA); and CA19-9 (ab3982) antibody (Abcam, Eugene, OR, USA).

## Measurement of the cAMP level

The cAMP levels in cells and EVs were determined by the cAMP-Glo Max Assay (Promega), in accordance with the manufacturer’s instructions. Cells on 96-well plates were treated with EVs for 1 h and washed in PBS. EVs in PBS were transferred to 96- well plates. For both cells and EVs, PBS with 20 mM MgCl2, 0.5 mM 3-isobutyl-1- methylxanthine, and 0.1 mM Ro 20-1724 (phosphodiesterase inhibitor) was added to inhibit cAMP degradation. Lysed samples were incubated with the provided inactive protein kinase A (PKA) for 20 min to activate PKA, luciferin was added, and the remaining adenosine triphosphate was quantified. The cAMP solution provided was used as the standard. Luminescence was analyzed using the GloMax Multi-Detection System (Promega).

## CA19-9 enzyme-linked immunosorbent assay

To determine the CA19-9 levels in serum, in EVs isolated from serum, or in EVs isolated from cell culture, the TM-CA 19-9 ELISA RUO Kit (DRG Instruments GmbH, Marburg, Germany) was used in accordance with the manufacturer’s instructions.

## Immunoprecipitation

Immunoprecipitation (IP) was performed using a CA19-9 (010-25901, Wako) or CD63 (012-27063, Wako) antibody and Protein A/G Magnetic Beads (BioVision, Mountain View, CA, USA). For IP-Western blotting, 10 μL of beads and 2 μg of CA19-9 or CD63 antibody were rotated for 1 h at room temperature, then washed twice with IP buffer (50 mM Tris [pH 7.5], 150 mM NaCl, 0.1% NP40, 1 mM ethylenediaminetetraacetic acid [pH 8.0], 0.25% gelatin, and 0.02% sodium azide). EVs (3 μg) were mixed with the antibody-conjugated beads overnight at 4°C using the HulaMixer Sample Mixer (Thermo Fisher Scientific); mixing comprised rotation at 10 rpm for 5 s in the orbital direction, 30 degrees for 5 s in the reciprocal direction, and 2 degrees for 3 s in vibration. Next, the beads were washed three times in IP buffer, incubated in 1× sample buffer for 15 min at room temperature, and subjected to electrophoresis. EVs (0.06 μg ~ 0.6 μg) were used as input for IP. For droplet digital PCR (ddPCR) and NanoSight analyses, 1 μL of beads and 5 μg of CA19-9 or CD63 antibody were rotated for 1 h at room temperature, washed four times in PBS, and rotated in PBS with 10% bovine serum albumin (pH 5.2) (Fraction Ⅴ) (Wako). EVs (3 μg) were mixed with the antibody-conjugated beads, rotated overnight at 4°C using the HulaMixer Sample Mixer as described above, and washed four times in PBS. For NanoSight analysis, EVs were eluted with 0.1 M Glycine-HCl Buffer Solution (pH 2.2) (Wako). Normal mouse IgG (140-09511, Wako) was used as a negative control for IP.

## RNA extraction from EVs and droplet digital PCR

RNAs in EVs were extracted using the Exosomal RNA Isolation Kit (Norgen Biotek, Thorold, Canada), followed by reverse transcription with SuperScript III First-Strand Synthesis SuperMix for qRT-PCR (Thermo Fisher Scientific), in accordance with the manufacturer’s instructions. Because of the low copy number of *KRAS* mRNA sequences extracted from EVs that had been immunoprecipitated using anti-CA19-9-conjugated beads, preamplification was necessary for ddPCR. Thus, 7.3 μL of cDNA template in a 12-μL reaction were subjected to PCR using LA Taq (TaKaRa Bio). The preamplification conditions were as follows: 1 min of denature at 95°C, followed by seven cycles of 94°C for 20 s and 55°C for 90 s, and finally 72°C for 5 min. The forward primer was 5ʹ-GTTGGATCATATTCGTCCAC-3ʹ; the reverse primer was 5ʹ-GCCTGCTGAAAATGACTGAA-3ʹ. ddPCR was performed using 5 μL of preamplified products, as previously described ^6^ and in accordance with the manufacturer’s instructions. Briefly, droplets containing the DNA template and TaqMan probes were generated using the QX200 Droplet Generator (Bio-Rad Laboratories). The ddPCR *KRAS* Screening Multiplex Kit, which contained buffers and probes for detecting wild-type *KRAS* and seven mutant *KRAS* alleles, was purchased from Bio-Rad Laboratories. Droplets were processed in a thermal cycler with 10 min of denature at 95°C, followed by 50 cycles of 94°C for 30 s and 55°C for 60 s, and finally 98°C for 5 min; each DNA sequence was amplified by TaqMan PCR in the droplets. Samples were processed in the QX200 Droplet Reader (Bio-Rad Laboratories), positive or negative droplets were detected based on the fluorescence intensity, and levels were corrected based on a Poisson distribution with QuantaSoft (Bio-Rad Laboratories).

## Immunofluorescence staining

For staining of β-actin, Panc-1 cells (wild type and Panc-1-ACTB-HiBiT) were incubated with a 1:300 dilution of β-actin antibody (#4970, Cell Signaling Technology), and for staining of CA19-9, Panc-1 and BxPC-3 cells in slide chambers were incubated with a 1:250 dilution of CA19-9 antibody (GTX635389, GeneTex, Irvine, CA, USA) overnight at 4°C; they were then incubated for 1 h at room temperature with a secondary antibody (1:300 dilution) conjugated to Alexa Fluor 488 (Thermo Fisher Scientific). After slides had been washed with PBS, they were mounted in Mounting Medium with DAPI (Vector Laboratories). Images were captured using the Olympus DP72 microscope digital camera system.

## Animal studies

Six-to-seven-week-old female BALB/c mice were purchased from CLEA Japan (Tokyo, Japan). EVs from HPNE, Panc-1-ACTB-HiBiT, and Capan-2 cells were intravenously injected into the lateral tail vein (5 × 10^10^/mouse), twice weekly for 4 weeks. The same volume of PBS was injected into control mice. The body weight of the mice was measured twice weekly. At 3 days after the final injection, the mice were euthanized, and the gonadal white adipose tissue (gWAT) was weighed. For hematoxylin and eosin staining, tissues were fixed in 10% formalin and stained at SeptSapie (Tokyo, Japan). Images were obtained using the Olympus DP72 microscope digital camera system. Lipid droplet size was analyzed using ImageJ software (National Institutes of Health). For Western blotting, gWAT was homogenized in lysis buffer and identical amounts of proteins were subjected to Western blotting.

## Immunohistochemistry

Unstained specimens from BALB/c mice were prepared at SeptSapie. Specimens were incubated at 60°C for 60 min, then deparaffinized; subsequently, the Histofine Mouse Stain Kit (Nichirei Bioscience, Tokyo, Japan) was used to stain HiBiT peptides with an HiBiT antibody; the Elite ABC Rabbit IgG Kit (Vector Laboratories) was used to stain laminin α4, laminin α5, and laminin γ1, in accordance with the manufacturer’s instructions. The following primary antibodies were used: HiBiT antibody (1:300 dilution; Promega); laminin α4 (1:200; 10465-1-AP) antibody (Proteintech); and laminin α5 (1:100; PA5-49930) and laminin γ1 (1:100; PA5-81992) antibodies (Thermo Fisher Scientific).

## Heatmap

A heatmap was generated based on the gene expression Z-scores in RefEx (https://refex.dbcls.jp/).

## Gene knockout

For gene knockout, lentiCRISPR v2 (Plasmid #52961, Addgene, Watertown, MA, USA) which constitutively express Cas9 protein and an arbitrarily predetermined guide RNA, was used. A guide RNA that specifically combined with the gene of our interest was inserted into lentiCRISPR v2. LentiCRISPRv2 hygro (Plasmid #98291, Addgene) was additionally used for double gene knockout; cloning was carried out in *Escherichia coli*. Lentiviruses were produced by transfection of the desired plasmid and lentiviral plasmid packaging mix (pPACK Lentivector Packaging Kit, System Bioscience, Palo Alto, CA,

USA) into 293T cells using the Effectene Transfection Reagent (Qiagen, Gelderland, Netherlands). After 48 h and 72h, supernatants containing the lentiviruses were collected and added to Panc-1, Maiapaca-2 cells and differentiated adipocytes. Cells were selected with puromycin (2 μg/mL) for 7 days or with hygromycin (200 μg/mL) for 7 days; Western blotting was used to confirm that the target gene had been knocked out.

## Flow cytometry

EVs (1.0 × 10^10^) were fluorescently labeled using the ExoSparkler Exosome Membrane Labeling Kit-Green (Dojindo), in accordance with the manufacturer’s instructions. Differentiated adipocytes were detached by pipetting, then mixed with labeled EVs in PBS for 30 min at room temperature using the HulaMixer Sample Mixer (Thermo Fisher Scientific) with orbital rotation at 10 rpm. After mixing, the adipocytes were washed twice with PBS and the fluorescence intensity was analyzed using the Guava EasyCyte Plus Flow Cytometry System (Guava Technologies, Hayward, CA, USA).

## Fluorescent labeling of EVs for NanoSight analysis

For collection of CA19-9-positive or CD63-positive EVs by IP, 15 μg of CA19-9 antibody (010-25901, Wako) and CD63 antibody (012-27063, Wako) were attached to 5 μL of Protein A/G Magnetic Beads (BioVision); EVs from patients with pancreatic cancer (5 μg) were mixed with antibody-conjugated beads overnight at 4°C using the HulaMixer Sample Mixer (Thermo Fisher Scientific), with rotation as described above. After beads had been washed with PBS, they were incubated three times (10 min each) with 0.1 mL/L Glycine-HCl Buffer Solution (pH 2.2) (Wako) to detach CA19-9-positive or CD63- positive EVs from beads. To prepare samples not subjected to IP, EVs were incubated

with 0.1 mL/L Glycine-HCl Buffer Solution (pH 2.2) for 30 min. After neutralization with Tris buffer (pH 12), samples (6.0 × 10^9^ particles) were mixed with primary antibodies in 100 μL of Can Get Signal Immunoreaction Enhancer Solution B (Toyobo) overnight at 4°C using the HulaMixer Sample Mixer. The following primary antibodies were used: 1 μg of integrin α6 (66906-1-Ig) and 1 μg of integrin β1 (66315-1-Ig) antibodies (Proteintech), and 0.8 μg of integrin αV (H-2: sc-376156) antibody (Santa Cruz Biotechnology, Dallas, TX, USA). Subsequently, Alexa Fluor Plus 405 (#A48255, Thermo Fisher Scientific) was added and rotated at the same speed for 1 h at room temperature. For removal of excessive antibodies and purification of EVs, qEV size exclusion chromatography (single 35 column) and automated fraction collection (Izon Science) were performed, in accordance with the manufacturer’s instructions. The purified EVs were visualized and counted using the NanoSight LM10 (Malvern Instrument), with or without a fluorescent filter.

## Electron microscopy by the NanoSuit method

To visualize EVs on the adipocyte surface and endocytosis of those EVs, scanning electron microscopy using the NanoSuit method was performed at NanoSuit Inc. (Shizuoka, Japan), which preserves wet samples in a high-vacuum environment for nondestructive examination ^7^. EVs were added to differentiated adipocytes for 4 h at 37°C; 2% NanoSuit Solution Type III was then added to the cultures. Images were captured by scanning electron microscopy.

## Statistical analysis

Statistical analysis and data visualization were performed using R statistical software. *P*-

values were determined by unpaired two-tailed *t*-tests; *P* < 0.05 was considered to indicate statistical significance. Correlations with the magnitude of lipolysis were estimated using Pearson’s correlation coefficient.

## Ethics approval

The animal experiments were approved by the Internal Ethics Committee for Animal Experimentation (approval number #H21-002) and were conducted in accordance with the Guidelines for the Care and Use of Laboratory Animals of the Graduate School of Medicine, University of Tokyo (Tokyo, Japan). For human clinical samples, written informed consent was obtained from the patients prior to participation. The study protocol was approved by the Ethics Committee of the University of Tokyo Hospital (approval number 11712).

## DATA availability

The data that support the findings of this study are available from the corresponding author upon reasonable request.

## SUPPLEMENTARY REFERENCES

1. Engle DD, Tiriac H, Rivera KD, et al. The glycan CA19-9 promotes pancreatitis and pancreatic cancer in mice. *Science* 2019;364: 1156-62.
2. Kalluri R, LeBleu VS. The biology, function, and biomedical applications of exosomes. *Science* 2020;367:eaau6977.
3. Biankin AV, Waddell N, Kassahn KS, et al. Pancreatic cancer genomes reveal aberrations in axon guidance pathway genes. *Nature* 2012;491:399-405.
4. Van Deun J, Mestdagh P, Agostinis P, et al. EV-TRACK: transparent reporting and centralizing knowledge in extracellular vesicle research. *Nature Methods* 2017;14:228-232.
5. Jung YJ, Kim HK, Cho Y, et al. Cell reprogramming using extracellular vesicles from differentiating stem cells into white/beige adipocytes. *Science Advances* 2020;6:eaay6721.
6. Kishikawa T, Otsuka M, Yoshikawa T, et al. Quantitation of circulating satellite RNAs in pancreatic cancer patients. *JCI Insight* 2016;1:e86646.
7. Takaku Y, Suzuki H, Ohta I, et al. A thin polymer membrane, nano-suit, enhancing survival across the continuum between air and high vacuum. *Proceedings of the National Academy of Sciences of the United States of America* 2013;110:7631-7635.

## SUPPLEMENTARY TABLES

| Change of body | | | | | BMI at the time of |
| --- | --- | --- | --- | --- | --- |
| patient | Age | Sex | Stage | weight | serum collection |
|  |  |  |  | at diagnosis |  |
| # 1 | 80 | Female | Ⅳ | 9.7 % loss | 17.6 |
| # 2 | 75 | Female | ⅠB | 4.9 % loss | 14.7 |
| # 3 | 54 | Female | ⅠA | ND | 32.1^※^ |
| # 4 | 74 | Male | Ⅳ | 2.6 % loss | 22.5 |
| # 5 | 46 | Male | Ⅲ | 5.4 % loss | 20.8 |
| # 6 | 72 | Male | Ⅳ | 9.8 % loss | 24.1^※^ |
| # 7 | 47 | Female | Ⅳ | ND | 17.4 |
| # 8 | 76 | Male | ⅠA | 5.2 % loss | 21.3 |
| # 9 | 70 | Male | Ⅳ | ND | 16.7 |
| # 10 | 62 | Male | Ⅳ | ND | 24.3^※^ |

BMI: Body Mass Index

ND: We have no data because information before diagnosis is unavailable.

※The patient’s edema or ascites were poorly controlled.

**Supplemental Table S1. Clinical characteristics of the patients included in this study.**

|  | Forward Primer | Reverse Primer |
| --- | --- | --- |
| C/EBPα | GAACACGAAGCACGATCAG | CCAAAACCAAAAGGAAAGGGAG |
| FABP4 | TGTGCAGAAATGGGATGGAAA | CAACGTCCCTTGGCTTATGCT |
| HSL | CTCAGTGTGCTCTCCAAGTG | CACCCAGGCGGAAGTCTC |
| GLUT4 | TCAACAATGTCCTGGCGGTG | TTCTGGATGATGTAGAGGTAGCGG |
| LAMA4 | GGAAAATAAGCGAGGCACCG | AGCCACAGAGGCAGAACCGA |
| LAMC1 | ATTTCAATCAACCGCTCT | GTTATGGACCTCCTTCGT |
| GAPDH | CAACTACATGGTTTACATGTTC | GCCAGTGGACTCCACGAC |

**Supplemental Table S2. Primers for qRT-PCR.**

**Suppelemtary Figure S1**

**A B**

MSC Adipocyte


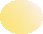

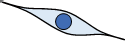

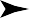


IBMX hAD-MSC

Insulin Dexamethasone

Indomethacin Day 0

Adipocyte

Day 21


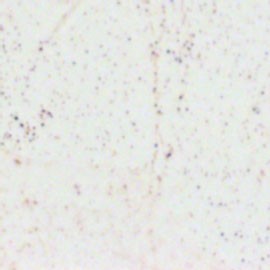

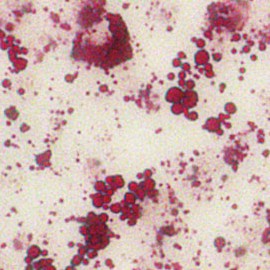


Scale bar, 20 μm

**C**

**

Oil red O μg/ml

3

2

1

0

MSC Adipocyte

**D** C/EBPα

**

40

Fold change

20

0

MSC Adipocyte

1,500

1,000

500

0

FABP4

**

MSC Adipocyte

100

50

0

HSL

**

MSC Adipocyte

GLUT4

**

15

10

5

0

MSC Adipocyte

**Supplementary Figure S1. Induction of mature human adipocytes from hAD-MSC.**

**(A)** Method for differentiation of human adipose-derived mesenchymal stem cells (hAD-MSCs) into adipocytes. **(B)** Images of Oil red

O-stained hAD-MSCs and differentiated adipocytes. Scale bar, 20 μm.um. Representative images of three independent experiments are shown.

**(C)** Oil red O levels in cells, as determined by colorimetric analyses. Data are means ± standard deviations (SDs) (n = 3). **P = 0.00020.

**(D)** Confirmation of adipocyte marker expression after differentiation. mRNA levels of C/EBPα, FABP4, HSL, and GLUT4 in hAD-MSCs and differentiated adipocytes were determined by reverse transcription (RT)-quantitative polymerase chain reaction (qPCR). Data are means ± SDs (n = 3). **P = 0.0028 (C/EBPα), **P = 0.00023 (FABP4), **P = 0.0021 (HSL), **P = 0.00065 (GLUT4).

**Supplementary Figure S2 A**


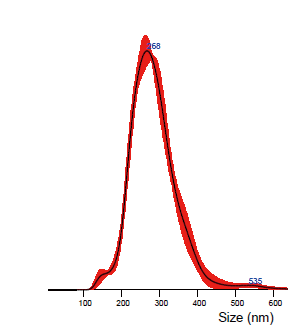

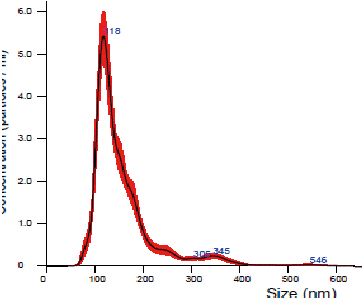

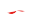

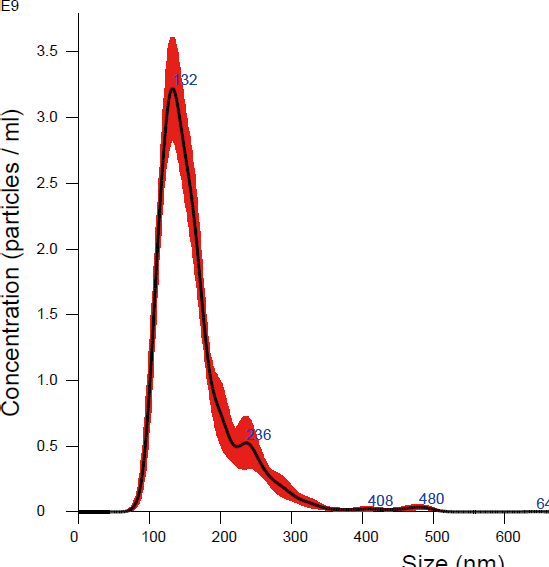


Panc-1

Miapaca-2

Capan-2

3.0

2.0

1.0

0

5.0

70

2.5

35

200 400 600

0

0

Size (nm)

200 400 600

Size (nm)

200 400 600

Size (nm)

Concentration (10⁹ particles/ml)

**B** TSG101 CD63

kDa 46

250


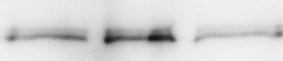

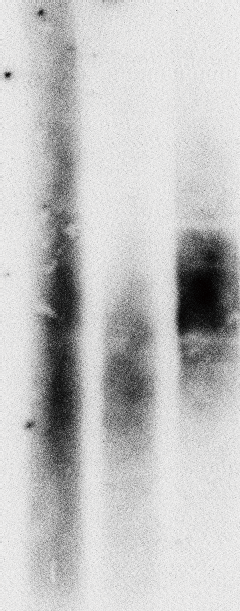


100

50

Panc-1

Miapaca-2 Capan-2

**Supplementary Figure S2. Characterization of EVs from the culture medium of pancreatic cancer cells.**

**(A)** Size distributions of EVs isolated from culture medium of the indicated cell lines. **(B)** Western blotting of exosome marker levels (CD63 and TSG101) in 0.8 μg of EVs from cell culture medium. Representative images of three independent experiments are shown.

**Supplementary Figure S3**

# A B


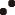


*

600

Glycerol (nM/h)

400

200

ns

300

Glycerol (nM/h)

200

100

0

Iso

# C

Iso + SQ

DAPI

Exosparkler

0

EV

Merge

EV + SQ

PBS


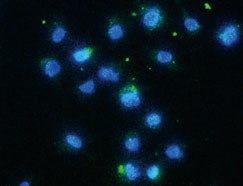

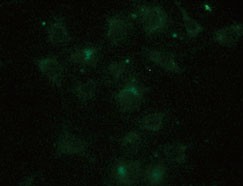

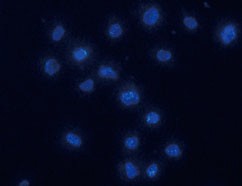

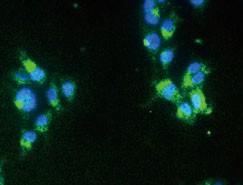

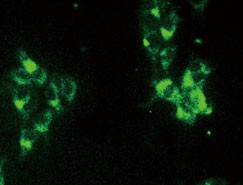

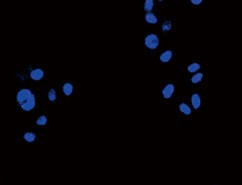

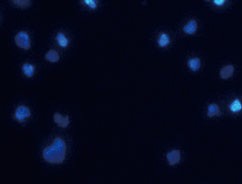

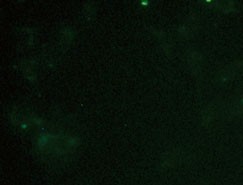

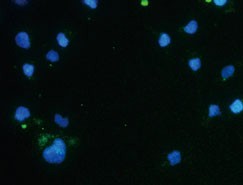


Panc-1 EV

Capan-2 EV

Scale bar, 30 μm

**Supplementalry Figure S3. Adipocyte endocytosis of EVs differs among cell lines.**

**(A)** Glycerol release from adipocytes treated with 10 μM isoproterenol (Iso) with or without 30 μM SQ22536 (SQ, adenylyl cyclase inhibitor) for 24 h. Data are means ± SDs (n = 3). **P* = 0.025. **(B)** Glycerol release from adipocytes treated with Panc-1 cell-derived EVs (1.5 μg) with or without 30 μM SQ22536 (SQ) for 24 h. Data are means ± SDs (n = 3). ns, not significant; *P* = 0.65. **(C)** Immunofluorescence images of adipocytes treated with PBS and EVs (1.5 μg) from Panc-1 and Capan-2 cell culture media for 5 h. Membranes of EVs were labeled with ExoSparkler (green). Nuclei were stained with DAPI (blue). Representative images of three independent experiments are shown. Scale bar, 30 μm. Statistical analyses were by Welch’s *t*-test.

**Supplementary Figure S4**

**A**


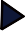


Panc-1


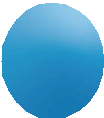

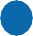


Cas9 + guideRNA

Donor DNA


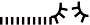


HiBiT

**B**

HiBiT


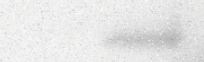

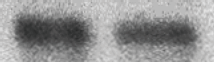


β-actin

**

Relative band

200

intensities

kDa 42

42

ACTB

HiBiT

5’ … gatgtggatcagcaagcaggagtatgacgagtccggcccctccatcgt

ccaccgcaaatgcttctaggcggactatgacttagttgcgttacaccctttc … 3'


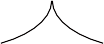


Red: Stop codon

Underline: PAM sequence

5'ggcggctgttcaagaagattagctagtcggact3'

0

WT ACTB

-HiBiT


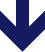


HiBiT

ACTB

**C**

Cell lysate

**

Relative luciferase value

150

100

50

0

WT ACTB

-HiBiT

**D**

20

EV

**

Relative luciferase value

10

0

WT ACTB

-HiBiT

**E** ACTB Nucleus Overlay WT

ACTB


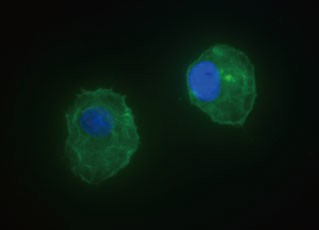

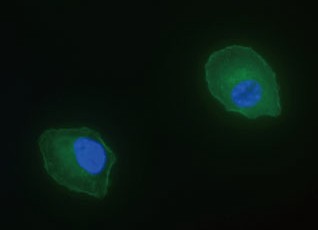

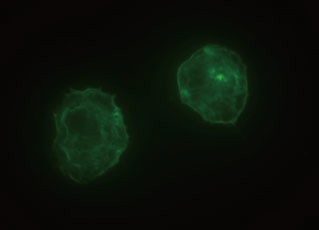

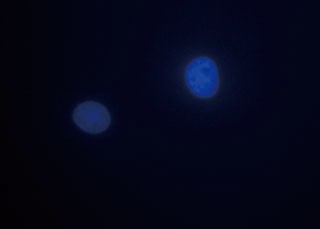

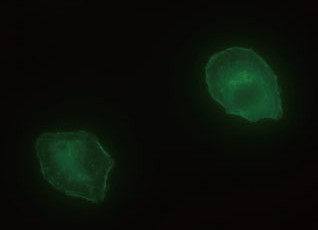

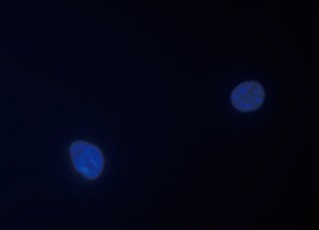


-HiBiT

Scale bar, 20 μm

**Supplementary Figure S4. Establishment of Panc-1 cells expressing HiBiT peptides fused to endogenous β-actin via knock-in gene editing.**

**(A)** Establishment of Panc-1 cells with HiBiT sequences downstream of the ACTB gene. HiBiT peptide sequences were inserted immediately before the stop codon of ACTB by Cas9-mediated gene editing; this yielded endogenous HiBiT peptide-conjugated β-actin. **(B)** β-actin-HiBiT in WT and ACTB-HiBiT cells, visualized by the HiBiT blotting system and by Western blotting with a β-actin antibody. Representative images from three independent experiments are shown. Relative band intensities of HiBiT normalized to β-actin are shown in the lower panel. Data are means ± SDs (n = 3). ***P* < 10^−4^. **(C, D)** Luciferase values of wild-type Panc-1 cells (WT) and Panc-1-ACTB-HiBiT cells (ACTB-HiBiT) (C), and of 10^9^ EVs from WT and ACTB-HiBiT cells (D). Data are means ± SDs (n = 4). ***P* < 10^−4^ (C), ***P* < 10^−5^ (D). Statistical analyses were by Welch’s *t*-test. **(E)** Immunofluorescence images of WT and ACTB-HiBiT cells. HiBiT knock-in did not influence the skeleton of the cells, determined by immunostaining of ACTB.

**Supplementary Figure S5**

# A

ITGB1 CD9

**

Relative band

intensities

1

0

# B

WT KO

kDa 135

21


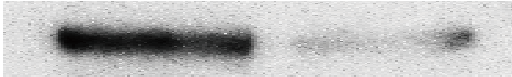

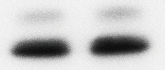


ITGA6 CD9

1

**

Relative band

intensities

0

WT KO

kDa 125

21


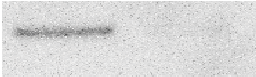

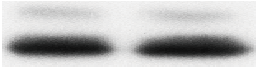


ITGAV CD9

1

**

Relative band

intensities

0

WT KO

kDa 135

21


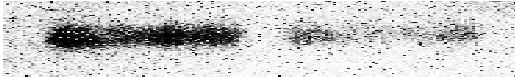

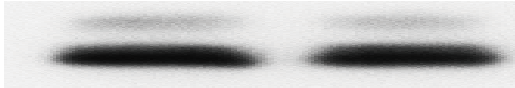


150


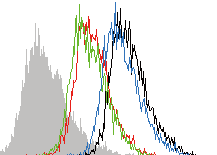


100

Count

50

0 10⁰

10¹ 10² 10³

104

Adipocyte

Adipocyte + Miapaca-2 EV (WT) Adipocyte + Miapaca-2 EV (ITGB1 KO) Adipocyte + Miapaca-2 EV (ITGA6 KO) Adipocyte + Miapaca-2 EV (ITGAV KO)

# C

300

Glycerol (nM/h)

200

100

0

Adhesion levels of EVs


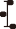

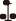

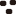


ns

*

*

WT B1 A6 AV

ITG knockout

**Supplementary Figure S5. Integrin β1 and integrin α6 on Miapaca-2 cell-derived EVs are required for lipolysis.**

**(A)** Western blotting to confirm the knockout of each integrin in Miapaca-2 cell-derived EVs. Representative images of three independent experiments are shown. Band intensities of each integrin relative to CD9 are shown in the lower panel. Data are means ± SDs (n = 3).

***P* = 0.00079 (ITGB1), ***P* = 0.0011 (ITGA6), ***P* = 0.00018 (ITGAV). **(B)** Flow cytometry analysis of adhesion to adipocytes among EVs derived from wild-type (WT), ITGB1-knockout (ITGB1 KO), ITGA6-knockout (ITGA6 KO), and ITGAV-knockout (ITGAV KO) Miapaca-2 cells. Representative images of three independent experiments are shown. **(C)** Glycerol release from human adipocytes treated with the indicated Miapaca-2 cell-derived EVs (1.5 μg) for 24 h. Data are means ± SDs (n = 4). **P* = 0.039 (B1 KO), **P* = 0.019 (A6 KO), *P* = 0.57 (AV KO).

Statistical analyses were by Welch’s *t*-test.

**Supplementary Figure S6**

# A

**

8,000

CA19-9 (U/ml)

4,000

Serum

**B** EV **C**

4,000

**

CA19-9 (U/ml)

CA19-9

2,000

kDa 250

150


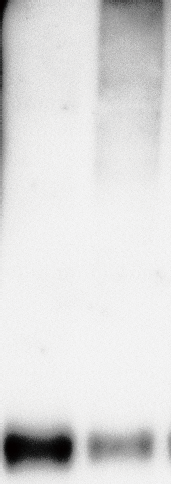


100

75

0

Healthy Cancer

0

Healthy Cancer

NS

HealthyCancer

# D E

**

**

ns


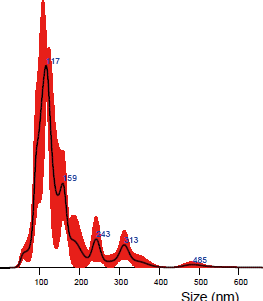

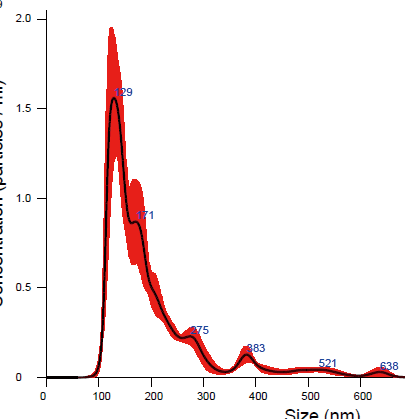

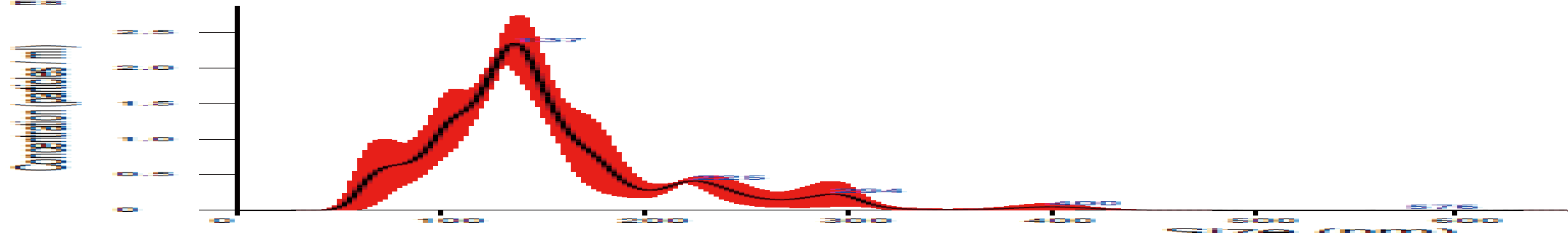


HPDE

0.2

0.1

0

200 400 600

Size (nm)

CA19-9 U / EV 1μg

0.3

Concentration (10⁹ particles/ml)

1.5

HPNE

2.0

1.0

BxPC-3

40

20

0 200

400

600 0

200

400

0

600

HPDE HPNE

Panc-1 Miapaca-2 BxPC-3

Capan-2

Size (nm) Size (nm)

**F** Cell lysate

kDa EV

250


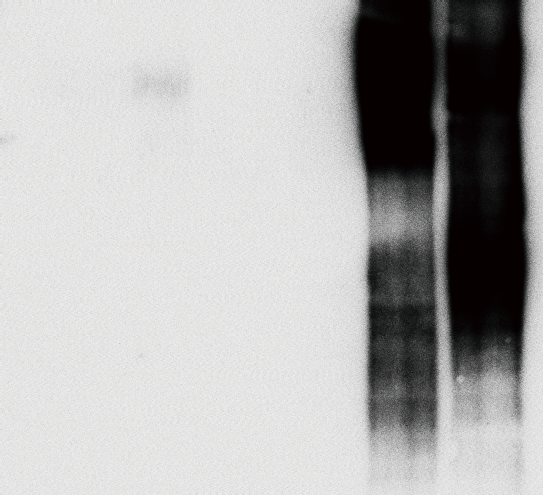

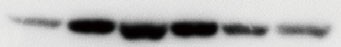

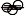

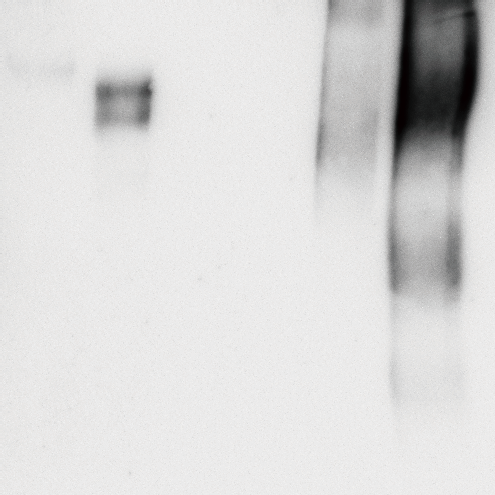

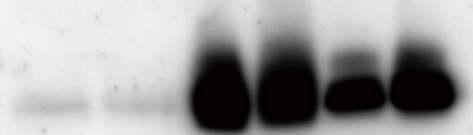

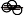


CA19-9

150

100

kDa 250

150

100

**G**

Panc-1

DAPI anti-CA19-9 Merge


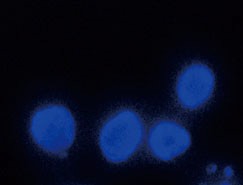

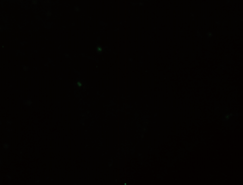

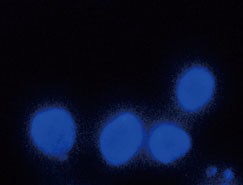

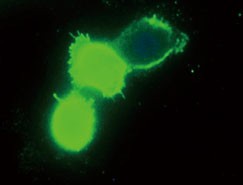

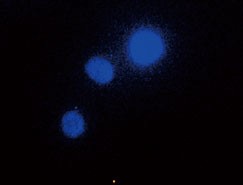

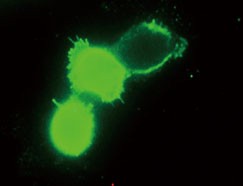


CA19-9

β-actin 100

Relative band intensities

0

75

42 CD9

20

0

HPDE HPNE

Panc-1 Miapaca-2 BxPC-3

Capan-2

HPDE HPNE

Panc-1 Miapaca-2 BxPC-3

Capan-2

75 BxPC-3

21

Scale bar, 10 μm

**Supplementary Figure S6. CA19-9 levels in EVs from the serum of patients with pancreatic cancer.**

**(A, B)** CA19-9 levels measured by enzyme-linked immunosorbent assays. CA19-9 levels in 1 mL of pooled sera (A) or EVs from 1 mL of pooled sera (B) from five healthy controls (Healthy) or five patients with pancreatic cancer (Cancer). Data are means ± SDs (n = 2 (A) and n = 4 (B)). ***P* = 0.00050 (A), ***P* < 10^−6^ (B). **(C)** Western blotting of CA19-9 in 0.2 μg of EVs from pooled sera of five healthy controls

(Healthy) or five patients with pancreatic cancer (Cancer). Representative images of three independent experiments are shown. NS, nonspecific band. **(D)** Size distributions of EVs from culture media of the indicated cell lines. **(E)** CA19-9 levels in EVs (1 μg) from normal human pancreatic ductal epithelial cells (HPDE and HPNE) and pancreatic cancer cell lines (Panc-1, Miapaca-2, BxPC-3, and Capan-2), as determined by enzyme-linked immunosorbent assays. EVs from normal human pancreatic ductal epithelial cells, Panc-1, and Miapaca-2 cells exhibit low levels of CA19-9. Data are means ± SDs (n = 4). ns, not significant. ***P* < 10^−5^. **(F)** Western blotting of CA19-9 levels in cell lysates and EVs (0.8 μg) from the indicated cell lines. Representative images of three independent experiments are shown. Band intensities of CA19-9 relative to β-actin (cell lysate) or CD9 (EVs) are shown in the lower panel. Data are means ± SDs (n = 3). **(G)** Immunofluorescence staining of CA19-9 (green) in Panc-1 (CA19-9-negative) and BxPC-3 (CA19-9-positive) cells. Nuclei were stained with DAPI (blue). Representative images of three independent experiments are shown. Scale bar, 10 μm. Statistical analyses were by Welch’s *t*-test.

**Supplementary Figure S7**

# A B

Huh7


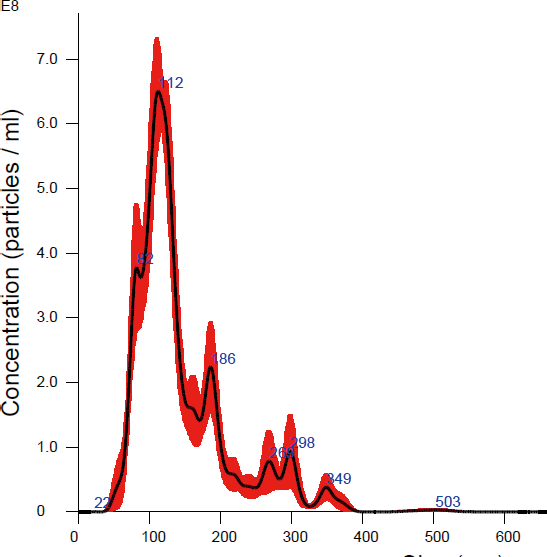

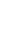


Concentration (10⁹ particles/ml)

0.6

CA19-9

0.3

IP: anti-CA19-9

IB: anti-CA19-9 / anti-AFP

kDa 250

150


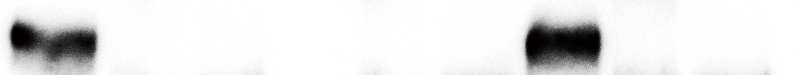

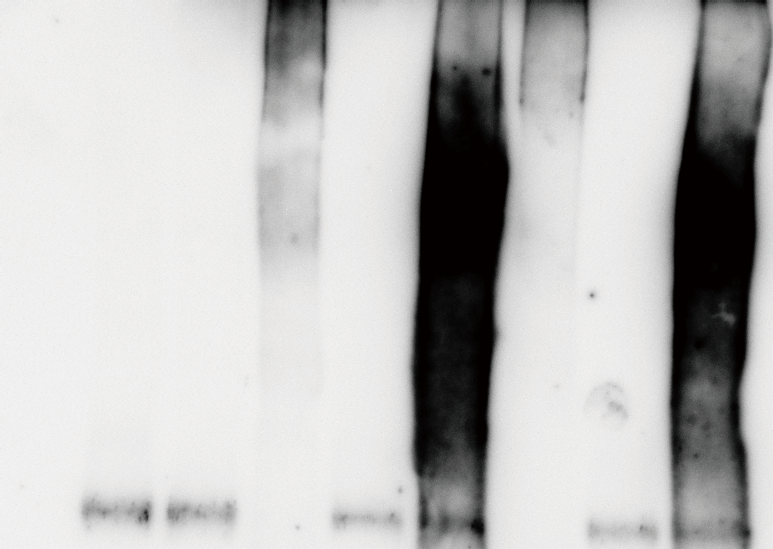


100

0 200 400 600 75

Size (nm)

Input IP (IgG)

IP (anti-CA19-9)

Input IP (IgG)

IP (anti-CA19-9)

Input IP (IgG)

IP (anti-CA19-9)

AFP 65

Huh7 EV Capan-2 EV

Huh7 EV+

Capan-2 EV

# C D

*KRAS* mution frequencies

1.0

0.5

ns

0.2

*KRAS* mution frequencies

0.1

**E**

0.6


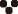


**

*KRAS* mution frequencies

0.4

0.2

0

Huh7 EV

Capan-2 EV

0

(-) (+)

Preamplificaion

0

Before IP

After IP

**Supplementary Figure S7. *KRAS* mutation frequency in pancreatic cancer-derived EVs.**

**(A)** Size distributions of EVs from culture medium of Huh7 cells. **(B)** EVs (3 μg) were immunoprecipitated using an anti-CA19-9 antibody, then subjected to Western blotting with anti-CA19-9 and anti-AFP antibodies. Isotype IgG was used as the control. Input was 2% EVs before immunoprecipitation. Huh7 cell (hepatocellular carcinoma cell)-derived EVs were AFP-positive and CA19-9-negative. Capan-2 cell (pancreatic carcinoma cell)-derived EVs were AFP-negative and CA19-9-positive. Representative images of at least three independent experiments are shown. **(C)** *KRAS* mutation frequencies (*KRAS* mut/WT ratio: ratio of mutant and wild-type KRAS RNA frequencies) in RNAs from Huh7

cell-derived EVs and Capan-2 cell-derived EVs, as determined by ddPCR. Huh7 has a wild-type *KRAS* gene, while Capan-2 has a heterozygous mutant *KRAS* gene. Data are means ± SDs (n = 3). **(D)** *KRAS* mutation frequencies in RNAs from pooled Huh7 cell- and Capan-2 cell-derived EVs at a ratio of 1:1, as determined by ddPCR. Preamplification did not affect the ratios. Data are means ± SDs (n = 3). ns, not significant;

*P* = 0.55. **(E)** *KRAS* mutation frequencies in RNAs from EVs before and after IP using an anti-CA19-9 antibody from a 1:1 mixture of Huh7 cell- and Capan-2 cell-derived EVs, as determined by ddPCR. Data are means ± SDs (n = 3). ***P* = 0.0080. Statistical analyses were by Welch’s *t*-test.
